# The Old and the New: A Comparison in Out-Patient Departments

**Published:** 1912-10-19

**Authors:** 


### THE OLD AND THE NEW.

### A Comparison in Out-patient Departments.

We have more than once referred to the urgent duty of every hospital that claims to possess an out-patient department to spare no means or trouble in securing in that department conditions such as are demanded by modern medical science on the one and by humanitarian hospital opinion on the other hand. The reform of out-patients is not a mere matter of sentiment; it is a question that has its practical side. From a purely business point of view a good out-patient department is a valuable asset. Therein, perhaps better than in the wards, the layman can actually estimate the importance of the work that is being carried on. When such work is done under adverse hygienic conditions, in faulty premises, and in an environment of filth and decay it rouses instead of sympathy a profound feeling of disgust with the management that allows such a state of things to exist.

In North-West London there has for several years existed an out-patient department that was, so far as its structural and hygienic conditions were concerned, a standing disgrace to the hospital with which it was associated. This was the Kentish Town casualty department of the combined Hampstead and North-Western Hospital. Housed in an old and gloomy building, it fulfilled, like the celebrated pump of the hygienist described by Mr. Maartens, all the requisite conditions for the spread of sepsis, and threw a terrible strain upon all those who worked in it and all those who were worked upon. Its ventilation was a puzzle to find; its exterior, coloured that particularly unattractive hue which the French graphically describe as "bullocks' blood," was as unattractive as its interior was faulty. No modern hospital worker having once traversed it would have hesitated in concluding that common sense and hygiene demanded its demolition. We know that the committee and the medical staff were fully aware of its shortcomings; we also know that, in spite of these internal miseries, good work was done, and that in a sense the department served its purpose. Indeed, we do not intend to animadvert upon the want of enthusiasm that allowed such a department to exist for so many years; our purpose in writing this article is rather to draw the attention of hospital workers to the fact that in Kentish Town there now exists two out-patient departments which admirably illustrate the essential difference between the old and the new. The Kentish Town Road building is closed but not demolished, and we invite readers who wish to test the comparison to step from the new department in Hamilton Place to the old one just a hundred yards farther off and investigate the differences for themselves.

The new out-patient and casualty department of the Hampstead General Hospital, built from Mr. Young's designs, was only opened a few weeks ago, and still possesses the architectonic bloom of a building fresh from the contractor's hands. Outside it is plain, almost to the verge of ugliness, for its terra-cotta brick and darker tile mouldings, with plain cement facings, are not beautiful. But the colour scheme is harmonious, and the tints are quiet and useful. The building is doubled storeyed, with a frontage in Hamilton Place, and within half a minute's walk of the Camden Town Tube Station and the tramway. Standing well away from the main thoroughfares, it is quiet. In some ways it is almost an ideal building, though its plan is open to criticism on some points. Briefly stated, it consists of a basement floor, with kitchen, porter's room, servants' dining room, steam and gas house, larders, and extra rooms; of a ground floor, which contains the out-patient and casualty departments; and of a first floor containing the matron's, nurses', and resident medical officer's rooms. Simplicity is everywhere the dominating note, but there is no indication that efficiency has been sacrificed to cheapness or undue economy. The rooms for the nurses and medical officer are comfortable and good, and perhaps the only fault in the first floor is that the matron or sister-in-charge has no separate sitting room, but is given a bed-sitting room only. An additional room should be provided for her so as to ensure absolute privacy. In the basement the visitor is at once struck by one omission—the want of adequate cupboard space. This is, indeed, a drawback which should, and can be, easily remedied in all the rooms. The lighting of the basement is necessarily poor, but has been well arranged when one considers the difficulties. The building is heated by gas and lit by electric light, but both installations are as yet on their trial, though the fixtures are of the best.

Naturally the important part is the ground floor. There are separate entrances for out-patients and casualties. The porter's office commands the entrance hall and waiting room, which is well lit by roof lighting and has a cement floor that is easily cleaned. From this central hall exits lead into the various examining and side rooms. On the left are the surgeon's room, with two small examination rooms, a good, well-lighted minor operating room, plain but excellent in design and construction, with a recovery room and a separate exit; a physician's room, and a fine large room, provided with dark room for ophthalmoscopic work, for the ophthalmic surgeon. Then comes a small room for the dental surgeon and two small observation wards, single bedded, with lavatory and sluice chambers attached. A short passage leads into the fairly large yard, destined ultimately for the ambulance house, at the farther end of which is a simple mortuary. Facing the porter's lodge at the end of the central hall is the dispensary, with adjacent store room, and a small waiting lobby, with exit into the street, for patients. On the right of the hall is a room for the inquiry officer, Miss Gleg, whose work promises to be increasingly difficult. Further forward are two double lavatories, one for men and the other for women. Then comes a very excellently designed isolation unit, complete with isolation room, lavatory, and separate exit into the street; next to it is the $x$-ray room, with its developing chamber, and, finally, the large, airy, well-lighted casualty room, with a separate examining room.

This brief description gives the essentials of the block, which we venture to think is one of the most interesting in London. Criticism will doubtless be directed mainly to the lavatories, which, in our opinion, have been badly placed, necessitating the erection of screens, and to the ventilation. When we inspected the building there were comparatively few patients, and there was a distinct lack of that *Menschenduft* which is so prominent in many out-patient departments. We noticed a most unfortunate fixture in the central hall—an open drinking fountain with attached cup; such a thing is utterly out of place in an out-patient department, and should be removed, since it only serves as a source of infection and contamination. The flooring in the side rooms and upper floor is of doloment, already, we noticed, looking worn and faded. We should have thought a plain linoleum on cement base would have served better.

These, however, are minor criticisms. The main point is that the Hampstead Hospital at last possesses an out-patient department of which it may justly be proud. Let us add, however, that to ensure the best work being done in it the authorities should provide an adequate nursing staff. The average number of out-patients exceeds one hundred per day, and the number of casualties is rapidly rising. Provision has been made for two resident medical officers, which is adequate, but there are only two nurses to help Miss Kitching, the sister-in-charge, on whose shoulders rest practically the whole work of housekeeping and superintending. This is a serious drawback, for the honorary medical staff has only the services of one nurse and the other is overworked in the casualty department. In our opinion the nursing staff should be doubled by the addition of a probationer and a trained nurse.

